# The prevalence of menstrual disorders in Iran: A systematic review and meta-analysis

**Published:** 2018-11

**Authors:** Reza Omani Samani, Amir Almasi Hashiani, Maryam Razavi, Samira Vesali, Mahroo Rezaeinejad, Saman Maroufizadeh, Mahdi Sepidarkish

**Affiliations:** 1 *Department of Epidemiology and Reproductive Health, Reproductive Epidemiology Research Center, Royan Institute for Reproductive Biomedicine, ACECR, Tehran, Iran.*; 2 *Pregnancy Health Research Center, Department of Obstetrics and Gynecology, School of Medicine, Zahedan University of Medical Sciences, Zahedan, Iran.*; 3 *Department of Obstetrics and Gynecology, Arash Women's Hospital, Tehran University of Medical Sciences, Tehran, Iran.*

**Keywords:** Menstruation disturbances, Amenorrhea, Dysmenorrhea, Menorrhagia, Oligomenorrhea

## Abstract

**Background:**

Understanding the prevalence of menstrual disorders has important implications for both health service planning and risk factor epidemiology.

**Objective:**

The aim of this review is to identify and collate studies describing the prevalence of menstrual disorders in Iran.

**Materials and Methods:**

Studies with original data related to the prevalence of menstrual disorders were identified via searching six electronic databases and reviewing citations. All abstracts or titles found by the electronic searches were independently scrutinized by two reviewers. The Meta-analysis was performed with a random effects model, considering the remarkable heterogeneity among studies. A total of 35 eligible epidemiological studies were included in this review.

**Results:**

Overall, the pooled prevalence of primary dysmenorrhea was 73.27% (95% CI=65.12-81.42). The mean proportion of women with oligomenorrhea was 13.11% (95.5%, 95% CI: 10.04-16.19). We identified 16 studies that reported polymenorrhoea with a random effect of pooled prevalence estimate of 9.94% (95% CI 7.33%-12.56%). The prevalence estimate of hypermenorrhea was 12.94% (95% CI 9.31%-16.57%). Overall prevalence of hypomenorrhea was 5.25% (95% CI 3.20%-7.30%), ranging from 0.9- 12.90%. Pooling six studies that reported estimates for menorrhagia, the overall prevalence was 19.24% (95% CI 12.78-25.69). Overall, 6.04% (95% CI: 1.99-10.08) of the women were shown to have metrorrhagia.

**Conclusion:**

This systematic review suggests that the average prevalence of menstrual disorders in Iran is substantial. It has been neglected as a fundamental problem of women's reproductive health. Diagnosis and treatment of these disorders should be included in the primary health care system of reproductive health.

## Introduction

Menstruation is a natural monthly occurrence during all reproductive life in healthy adolescent girls, and non-pregnant and pre-menopausal adult women (1). Menstrual bleeding is discharge of the inner lining of the uterus following the reduction of secreted hormones from ovaries and shrinkage of corpus luteum (2). The menstrual pattern is indicative of the health status of women. It usually occurs in regular intervals. Abnormal uterine bleeding is due to multiple hemorrhagic manifestations in the menstrual cycle without any pathologic cause or specific disease. It usually indicates anovulatory; abnormal bleeding, however, is not resulting from anovulation (3). The bleeding is more observed in women who aged either younger or older in reproductive life (4). It is known that one of the most common cause of abnormal uterine bleeding in all ages is hormonal disorders, but in adolescents and young adults other factors, including pregnancy-related bleeding, exogenous hormones, endocrine disorders (hypothyroidism, diabetes, etc.), neurological anorexia, obesity, uterine myoma and infections, are considered more effective causes (5). 

In a 2 yr national survey, it was found that of the total 20 million visits to medical clinics due to genital diseases, 19.1% of cases was attributed to menstrual cycle disorders and abnormal uterine bleeding is responsible for the cause of 25% of gynecological surgeries (6).

It is clear that every woman has experienced a history of menstrual problems in her lifetime. Irregular menstrual cycles and menstrual dysfunction can be accompanied with a remarkable effect on the mood of young girls or women and their everyday social activities (7). Yet, although investigations in different developing countries show that women are concerned by menstrual disorders, much less attention is paid to menstrual health and menstrual disorders as a health priority, especially in developing countries (8). A strategy is required for improving the quality of services provided to women with menstrual problems. It is depended on awareness of population about the prevalence of menstrual morbidity. 

This systematic review and meta-analysis article attempted to estimate the prevalence of menstrual morbidity in Iranian population of women aged 18-45 yr.

## Materials and methods

**Sources and study selection**

Studies were selected for this review based on predefined criteria. Observational studies in the form of cohort (prospective or retrospective) and cross-sectional were considered acceptable for inclusion. We excluded the following studies: 1) interventional or experimental studies, 2) case control studies, 3) ecologic studies, and 4) case series or case report studies. The most ‘‘informative version’’ of the study was included if multiple publications presented identical data. Studies published in a language other than English were translated. Then, relevant papers were included. The population of interest was women aged between 9 to 45 yr. The primary outcome of interest was prevalence of menstrual disorders as following:

1. Primary dysmenorrhea occurring cramping pain in the lower abdomen just before or during menstruation in the absence of other diseases such as endometriosis (9).
2. Oligomenorrhea: infrequent and irregular menstrual periods (more than 35 days without menstruation) (10).
3. Polymenorrhea: the occurrence of menstrual cycles at frequency that is higher than normal (less than 21 days) (11).
4. Hypermenorrhea: prolonged menstrual bleeding at regular intervals (more than 8 days) (12).
5. Hypomenorrhea: less blood flow or the duration of menses less than two days (13).
6. Menorrhagia: occurring excessive uterine bleeding at regular intervals (greater than 80 mL of blood loss per cycle) (13).
7. Metrorrhagia: uterine bleeding at irregular intervals, particularly between the expected menstrual periods (14).

**Search methods**

Electronic searches using the MeSH terms were conducted in international and national electronic databases as following: Medline, Embase, Scopus, Web of Science, Google Scholar, Magiran, SID, and Iranmedex. We checked the citation lists of relevant publications, review articles and included studies. For additional relevant citations, we hand searched references of identified selected articles. We also contacted experts and specialists in the field for possible unpublished research on the topic and additional relevant citations. A search strategy was carried out based on the following terms: “menstruation disturbances”, “dysmenorrhea”, “prevalence”, “metrorrhagia”, “amenorrhea”, “oligomenorrhea”, “menorrhagia”, and “Iran”.

**Data extraction and management**

All abstracts or titles found by the electronic searches were independently analyzed carefully by two reviewers (blinded to study authors, institutions, journal name, volume and page numbers) and any disagreement between reviewers was resolved by a third party. Data were extracted according to study characteristics, including study size, setting, sampling, response rate, and measurement of exposure by using a data extraction form designed and pilot tested by the authors. The quality of all eligible studies was assessed using Newcastle-Ottawa Scaling for cross-sectional studies (15). The evaluation of the studies was based on the following domains: the selection of the study groups; the comparability of the groups; and the ascertainment of outcome.

**Statistical analysis**

In order to estimation of pooled prevalence of menstrual disorders, we extracted either numerator and denominator, or prevalence and denominator, or prevalence and standard error, or prevalence and 95% confidence intervals. Numerator and denominator could then be calculated from any of these combinations. Statistical heterogeneity among the studies was assessed by visual inspection of forest plots, Cochrane Q test and I^2^ statistic. Statistical heterogeneity was considered substantial if the p-value was less than 0.1 or I^2^ value exceeded 50%. A separate random-effects model was constructed for each disorder using the DerSimonian-Laird weighting method, which incorporates between-study variability into the calculations. 

In this model, we assume that each study estimates a study-specific true effect μi. Interest then lies in estimating the mean μ=E (μi) and variance Var (μi) =τ2 of these true effect sizes across the population of potential studies. In a random-effects meta-analysis, the observed heterogeneity in the estimates μ^i is attributed to two sources: 1) between-study heterogeneity in true effects, and 2) within-study sampling error. We assessed the probability of publication bias with Egger’s test, with p<0.10 considered representative of statistically significant publication bias. All statistical analyses were performed using STATA version 13.0 (Stata Corp, College Station, TX, USA).

## Results

**Study identification**

**Figure 1:**
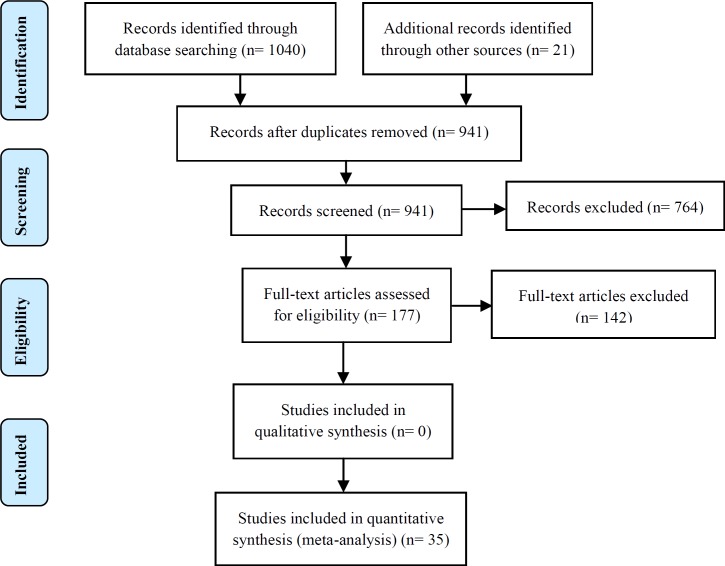
Flow diagram of the literature search for studies included in meta-analysis

Electronic searching retrieved 1061 citations from aforementioned databases, and 120 were excluded due to duplicate publications. After the process of reading the titles and abstracts, 764 publications were excluded as clearly ineligible, leaving 177 for further review. Of those, 35 fulfilled all inclusion criteria. A further 142 were excluded because they had inappropriate outcome measures (n=68), had inappropriate patient populations (n=53), and did not examine the appropriate disease state (n=21). Figure 1 shows the results of the literature search and selection process based on the PRISMA flow chart for systematic reviews. 

**Description of studies**

Tables I provide descriptive details of the included studies. These studies were published between 2001 and 2015. The sample size of included articles varied from 70 to 3200, with a total of 21344 cases. The largest study, a school-based study undertaken in Fars (Shiraz), screened more than 3200 students (16). Articles originated from 21 provinces, with Tehran contributing more studies than any other provinces (n=5). There were 27 school-based studies, 19 studies in high school, and 8 studies in university. Also 5 studies were conducted in general population. In some of the studies, the population was restricted to a specific subgroup: nurses screened for possible menstrual disorders (n=1), blind girls (n=1), and female workers in the packaging units of the pharmaceutical factory (n=1). Quality was assessed of all 35 studies according to the criteria shown in table II. The maximum score for quality according to these criteria is 4. The actual scores ranged from 4-9. Majority of studies classified as satisfactory and good quality. Twenty six studies presented data on the prevalence of primary dysmenorrhea, 20 studies on Oligomenorrhea, 16 studies on polymenorrhoea, 8 studies on hyper-menorrhea, 11 studies on hypomenorrhea, 11 studies on menorrhagia, 6 studies on metrorrhagia, and 5 on secondary amenorrhea.

**Figure 2:**
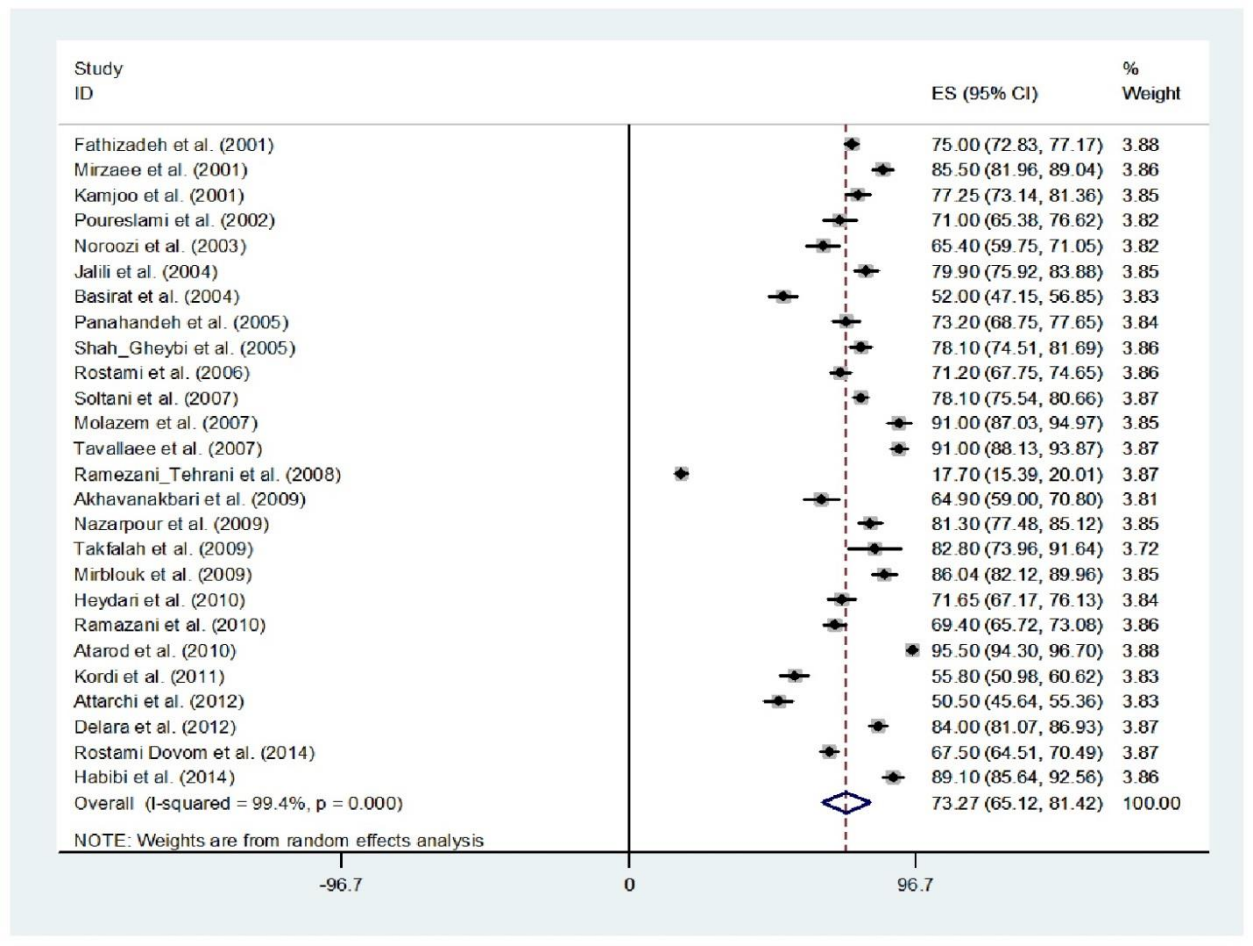
Forest plot showing prevalence of primary dysmenorrhea in Iran

Overall, the pooled prevalence of primary dysmenorrhea was 73.27% (95% CI=65.12-81.42). This analysis revealed significant heterogeneity across studies (Q=4097.93, d.f.=25, p<0.001 and I2=99.4%). The lowest and highest prevalence of primary dysmenorrhea was reported by Rostami-Dovom and his colleagues (17) in four provinces (Qazvin, Golestan, Kermanshah, and Hormozgan) (17.7%, 95% CI: 15.39-20.01) and Atarod and his colleagues (18) in Mazandaran (Sari) (95.5%, 95% CI: 94.3-96.7) (Figure 2). There was no evidence of publication bias found by the Egger test (p=0.129).

**Figure 3:**
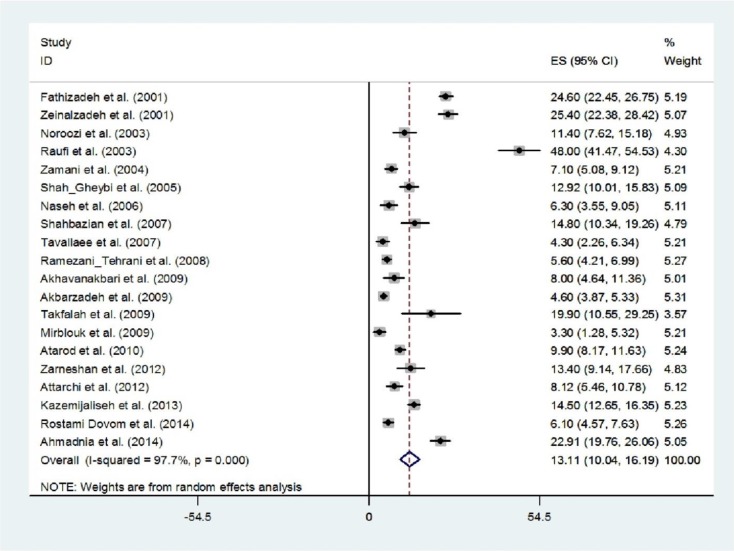
Forest plot showing prevalence of oligomenorrhea in Iran

The mean proportion of women that they had oligomenorrhea was 13.11% (95.5%, 95% CI: 10.04-16.19). The results of Cochran’s Q test and I^2^ statistics indicated substantial heterogeneity among the included studies (Q=810.54, d.f.=19, p<0.001 and I^2^=97.7%). As seen in figure 3, the highest prevalence of oligomenorrhea was reported by Raufi and his colleagues (19) in Fasa (south of Iran) (48%, 95% CI: 41.47-54.53). There was an evidence of publication bias found by the Egger test (p=0.003).

**Figure 4:**
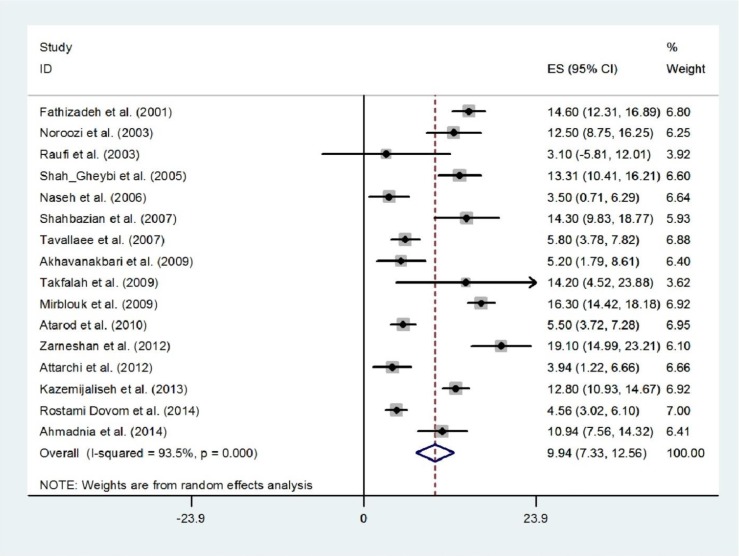
Forest plot showing prevalence of polymenorrhea in Iran

**Figure 5:**
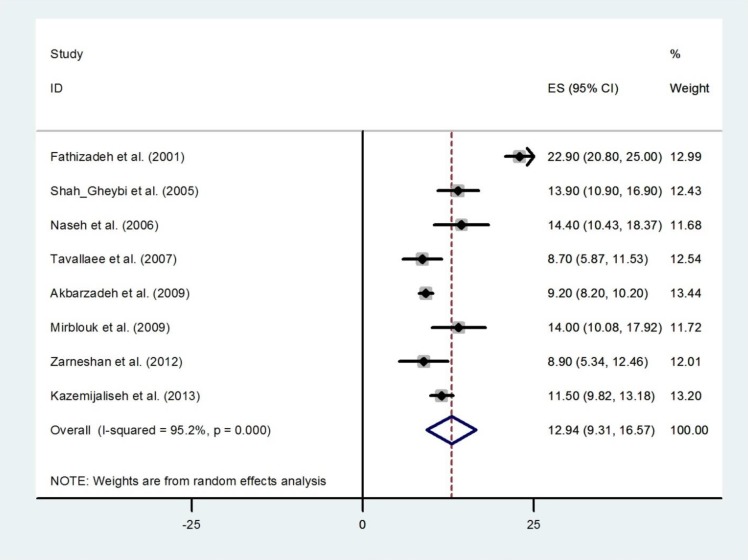
Forest plot showing prevalence of hypermenorrhea in Iran

**Figure 6:**
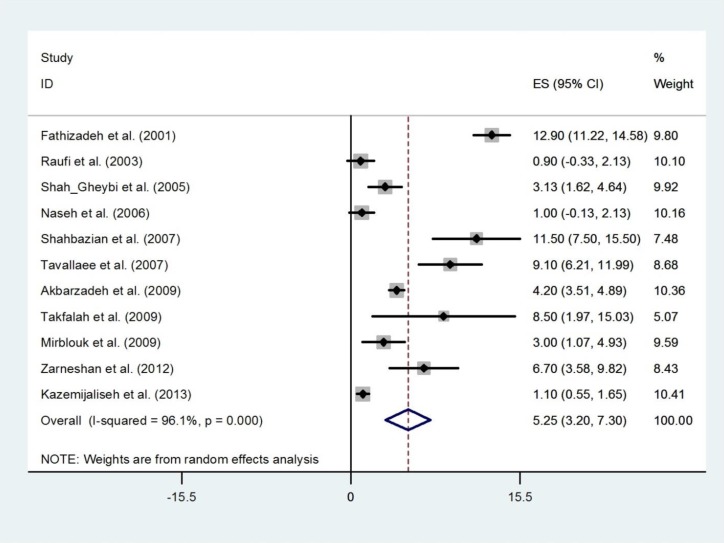
Forest plot showing prevalence of hypomenorrhea in Iran

We identified 16 studies that reported polymenorrhoea with a random effect of pooled prevalence estimate of 9.94% (95% CI 7.33%-12.56%) (Figure 4). The prevalence estimates ranged from 3.1% to 19.1% and there was substantial heterogeneity among those estimates (Q=230.38, d.f.=15, p<0.001 and I^2^=93.5%). There was no evidence of publication bias found by the Egger test (p=0.486). We identified 8 studies that reported hypermenorrhea in 7,868 women (Figure 5). The prevalence estimates ranged from 8.7% to 22.9%. The random effects of pooled prevalence estimate was 12.94% (95% CI 9.31%-16.57%) (Q=144.98, d.f.=7, p<0.001 and I^2^=95.2%). Overall prevalence of hypomenorrhea was 5.25% (95% CI 3.20%-7.30%), ranging from 0.9% in Fars (Fasa) (19) to 12.90% in Tehran (20). There was substantial heterogeneity among studies (Q=256.52, d.f.=10, p<0.001 and I^2^=96.1%) (Figure 6). There was no evidence of publication bias found by the Egger test (p=0.379).

**Figure 7:**
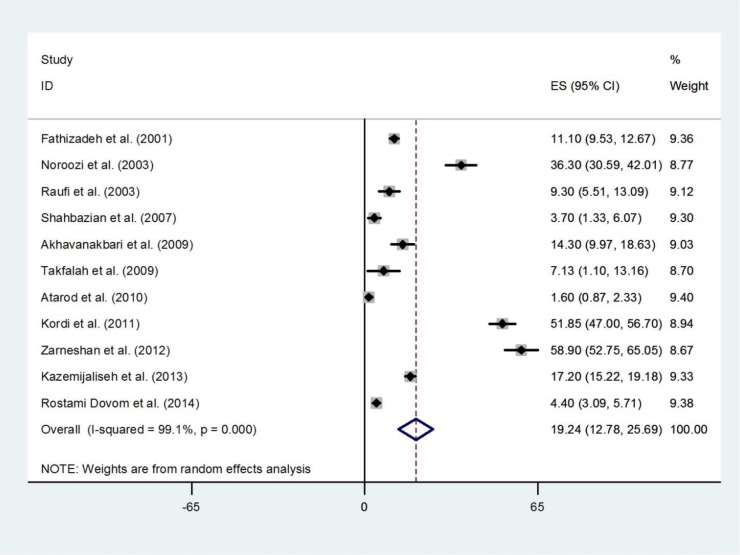
Forest plot showing prevalence of menorrhagia in Iran

**Figure 8:**
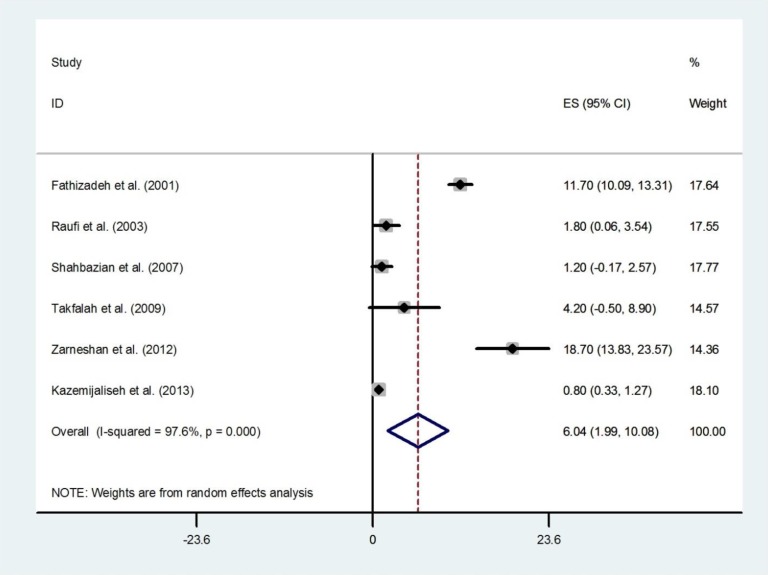
Forest plot showing prevalence of metrorrhagia in Iran

Pooling six studies that reported estimates for menorrhagia, the overall prevalence was 19.24% (95% CI 12.78-25.69) (Figure 7). The prevalence was highest in East Azerbaijan (58.9%, 95% CI 52.75-65.05) (21), and lowest in Mazandaran (1.6%, 95% CI 0.87-2.33) (18). There was an evidence of publication bias found by the Egger test (p=0.005). Overall, 6.04% (95% CI: 1.99-10.08) of the women were shown to have metrorrhagia. The results of Cochran’s Q test and I2 statistics indicated substantial heterogeneity among the included studies (Q=211.19, d.f.=5, p<0.001 and I^2^=97.6%) (Figure 8). There was no evidence of publication bias found by the Egger test (p=0.164).

**Figure 9:**
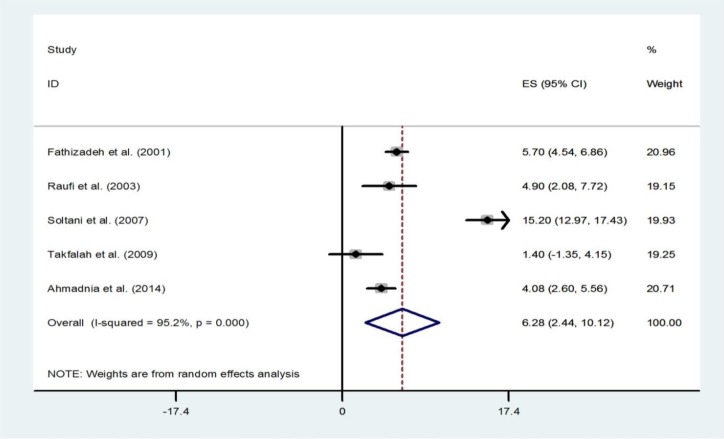
Forest plot showing prevalence of primary amenorrhea in Iran

A total of five study populations (3516 participants) provided data for the prevalence of secondary amenorrhea. Overall prevalence of secondary amenorrhea of these five studies was 6.28% (95% CI: 2.44-10.12; I2=95.2%; p<0.001) (Figure 9). There was no evidence of publication bias found by the Egger test (p=0.825). The number of studies that reported prevalence of primary amenorrhea was insufficient to statistically to calculate the pooled prevalence. Only one study of primary amenorrhea prevalence has been performed, in Mazandaran. This study was conducted from 2009 through 2010 in 1140 high school girls. The prevalence of primary amenorrhea was 1.2% (95% CI: 0.57-1.83) (18).

**Table I:** Description of the population and sample in the selected studies

**Study publication year**	**Province (city)**	**Participants**	**Sample size**	**Mean age (range)**	**Sampling**
Fathizadeh (2001) (20)	Tehran (Tehran)	High school student	1536	15.2 ± (-) (14-17)	Multistage sampling
Kamjoo (2001) (22)	**Hormozgan** **(Bandar abbas)**	College student	400	- (18-27)	Not mentioned
Mirzaee (2001) (23)	**Kerman** **(Rafsanjan)**	High school student	380	15.8 ± (3.8) (14-18)	Multistage sampling
Zeinalzadeh (2001) (24)	Mazandaran (Babol)	High school student	800	16.85 ± (2.5) (14-19)	Cluster sampling
Poureslami (2002) (25)	**Alborz** **(Karaj)**	High school student	250	- (15-18)	Simple random sampling
Noroozi (2003) (26)	**Bushehr** **(Bushehr)**	College student	272	- -	Convenience sampling
Raufi (2003) (19)	**Fars** **(Fasa)**	Women	225	- (15-51)	Convenience sampling
Basirat (2004) (27)	Mazandaran (Babol)	High school student	408	16.3 (1.15) (14-19)	Multistage sampling
Jalili (2004) (28)	Kerman (Sirjan)	High school student	390	18.24 (0.5) (17-18)	Multistage sampling
Zamani (2004) (29)	Kerman (Jahrom)	High school student	618	17.1 ± (2) (15-20)	Multistage sampling
Panahandeh (2005) (30)	Gilan (Rasht)	College student	380	21.3 (2) (18-27)	Multistage sampling
Shahgheybi (2005) (31)	Kurdistan (Sanandej)	High school student	511	- (17-18)	Systematic sampling
Naseh (2006) (32)	South khorasan (Birjand)	High school student	300	21.1 ± (2.1) (12-18)	Multistage sampling
Rostami (2006) (33)	Khuzestan (Masjed Soleiman)	High school student	660	(15-18)	Not mentioned
Molazem (2007) (34)	**Kohgiluyeh and Boyer-Ahmad**	High school student	200	15.3± (1.2) -	Multistage sampling
Shahbazian (2007) (35)	Khuzestan (Ahvaz)	High school student	244	13.6 ± (0.72) (12-16)	Cluster sampling
Soltani (2007) (36)	Hamadan (Hamadan)	High school student	1000	- (12-15)	Cluster sampling
Tavallaee (2007) (37)	**Tehran** **(Tehran)**	25-30 women	381	29.5 ± (6)	Stratified random sampling
Ramezani-Tehrani (2008) (17)	Four provinces( Qazvin, Golestan, Kermanshah, Hormozgan)	Women	1047	33.2±(7.7) -	Multistage sampling
Akbarzadeh (2009) (16)	Fars (Shiraz)	High school student	3200	- (14-18)	Not mentioned
Akhavanakbari (2009) (38)	West Azarbayjan (Ardabil)	College student	251	21.53 ± (2.2) (18-32)	Convenience sampling
Mirblouk (2009) (39)	Gilan (Rasht)	Nurses	301	-	Convenience sampling
Nazarpour (2009) (40)	**Tehran** **(Tehran)**	College student	400	- (18-22)	Convenience sampling
Takfalah (2009) (41)	Gilan (Rasht)	Blind girls	70	13.1 ± (0.8) (9-18)	Census
Atarod (2010) (18)	Mazandaran (Sari)	High school student	1140	15.4 ± (2.2) (14-18)	Cluster sampling
Heydari (2010) (42)	**Khuzestan** **(Ahvaz)**	College student	388	20.7 ± (1.8) (18-30)	Multistage Sampling
Ramazani (2010) (43)	Isfahan (Isfahan)	College student	601	20.86 ± (0.9) (18-30)	Multistage sampling
Kordi (2011) (44)	Khorasan Razavi (Mashhad)	High school student	407	- (14-16)	Multistage sampling
Attarchi (2012) (45)	**Tehran** **(Tehran)**	Female workers in the packaging units of the pharmaceutical factory	406	31.3 ± (4.9) (22-43)	Convenience sampling
Delara (2012) (46)	**Razavi Khorasan** **(Mashhad)**	High school student	602	15.78 )1.06) (14-19)	Not mentioned
Zarneshan (2012) (21)	East Azarbayjan (Tabriz)	High school student	246	20±(1.91) -	Convenience sampling
Kazemijaliseh (2013) (47)	Tehran (Tehran)	Women	1393	37.7 ± (11) (15-49)	Multistage sampling
Ahmadnia (2014) (48)	Zanjan (Zanjan)	High school student	685	14.5 ± (1.2) (11-18)	Not mentioned
Habibi (2014) (49)	**Isfahan** **(Isfahan)**	College student	311	20.69 (1.56) (18-27)	Not mentioned
Rostami-Dovom (2014) (17)	Four provinces (Qazvin, Golestan, Kermanshah, Hormozgan)	Women	941	32.9 ± (7.6) (18-45)	Multistage sampling

If one or more items are missing in the paper, these items were not presented in this table.

**Table II:** Results of the critical appraisal of the included studies

**Study (first author)**	**Selection of the study groups**				**Comparability of the groups**	**Ascertainment of outcome**		**Score**	**Quality**
	Representativeness of the sample	Sample size	Non-respondents	Ascertainment of exposure	Based on design and analysis	Assessment of outcome	Statistical test		
Fathizadeh, 2001 (20)	*			**		**	*	6	Satisfactory
Kamjoo, 2001 (22)	*	*	*	*		*	*	6	Satisfactory
Mirzaee, 2001 (23)	*	*		**		**	*	7	Good
Zeinalzadeh, 2001 (24)	*			*		*	*	4	Unsatisfactory
Poureslami, 2002 (25)	*	*	*	**		**		7	Good
Noroozi, 2003 (26)	*	*	*	*		*	*	6	Satisfactory
Raufi, 2003 (19)				*		*		2	Unsatisfactory
Basirat, 2004 (27)	*			*		*	*	4	Unsatisfactory
Jalili, 2004 (28)	*	*		**		**	*	7	Good
Zamani, 2004 (29)	*	*	*	*		*	*	6	Satisfactory
Panahandeh, 2005 (30)	*			**		**	*	6	Satisfactory
Shahgheybi, 2005 (31)	*			**		**	*	6	Satisfactory
Naseh, 2006 (32)	*			*		*	*	4	Unsatisfactory
Rostami, 2006 (33)	*	*	*	**	*	**	*	9	
Molazem, 2007 (34)	*	*		**		**	*	7	Good
Shahbazian, 2007 (35)	*	*	*	*		*	*	6	Satisfactory
Soltani, 2007 (36)	*	*		*		*	*	5	Satisfactory
Tavallaee, 2007 (37)	*		*	**		**	*	7	Good
Ramezani-Tehrani, 2008 (33)	*	*	*	**	*	**	*	9	Very Good
Akbarzadeh, 2009 (16)	*	*	*	*	*	*	*	7	Good
Akhavanakbari, 2009 (38)	*	*		**		**	*	7	Good
Mirblouk, 2009 (39)	*		*	*		*	*	5	Satisfactory
Nazarpour, 2009 (40)	*			**		**	*	6	Satisfactory
Takfalah, 2009 (41)	*	*		**		**	*	7	Good
Atarod, 2010 (18)	*	*	*	*		*	*	6	Satisfactory
Heydari, 2010 (42)	*	*		**		**	*	7	Good
Ramazani, 2010 (43)	*	*	*	*		*	*	6	Satisfactory
Kordi, 2011 (44)	*	*	*	**		**	*	8	Good
Attarchi, 2012 (45)	*			*		*	*	4	Unsatisfactory
Delara, 2012 (46)	*	*	*	*		*	*	6	Satisfactory
Zarneshan, 2012 (21)	*			**		**		5	Satisfactory
Kazemijaliseh, 2013 (47)	*	*	*	**	*	**	*	9	Very good
Ahmadnia, 2014 (48)	*	*	*	*		*	*	6	Satisfactory

## Discussion

This is the first comprehensive review that investigated the prevalence of all types of menstrual disorders in Iran. This review of the literature revealed that menstrual disorders are more prevalent in Iran. Of menstrual dysfunction, the prevalence of primary dysmenorrhea was 73.27%. Different prevalence of primary dysmenorrhea has been reported in the literature. In a systematic review by WHO, geographical distribution of primary dysmenorrhea was investigated and it was indicated that the rate of primary dysmenorrhea was 16-81% (50). Harlow and Campbell in 2004 conducted a review of the studies in developing countries and indicated that the prevalence of dysmenorrhea is between 15-68% (10). The results of the present review are in agreement with the mentioned studies that primary dysmenorrhea is common complaints. It is worth noting that the reported prevalence of primary dysmenorrhea in Iran was more than the rate reported from literature in developing countries. The difference can be due to lack of a standard tool for measuring the severity of dysmenorrhea, various definitions from this complaint in studies and populations investigated with different age groups. The results of this study confirmed the findings of previous studies that the prevalence of primary dysmenorrhea was higher in the early years after menarche and decreases with increasing age of women (51, 52). Most of studies included in the review investigated young girls and women who were at high school or college age and from every five women in reproductive age, three or four women were suffering from primary dysmenorrh. This disorder can be accompanied with decreasing everyday social activities of these women and other reproductive morbidities in them (3). Hence, clinical examination should be performed those sufferers from chronic and prolonged menstrual pain. In developing and less developed countries, reproductive health of women permeates with social, cultural and lifestyle, and we found fair/poor self-rated of the disorders and much less underestimated reports (33, 53). For reproductive health improvement of these women, gynecological examination is required to detect and diagnose adverse consequences. 

Our findings of hypomenorrhea and menorrhagia prevalence were 12.94% and 19.24%, respectively. In a review in studies on developing countries by Harlow, the frequency of abnormal uterine bleeding has been reported between 5% and 15% and then the prevalence increased with advancement of age (10). Few studies investigated the burden of abnormal uterine bleeding in developing countries. However, all of them reported the prevalence of 15% to 20% (54). Our findings of the rates of hypomenorrhea and menorrhagia are comparable to the report by Harlow (10). Differences in the frequency of menstrual bleeding in the existing studies can be due to several reasons. In some studies, the prevalence of the bleeding is measured based on face-to-face interviews or using self-reported questionnaires; while in some other studies, standard tools such as pictorial blood assessment chart or objective methods such as alkaline hematin are used (55). Abnormal bleeding may not be accompanied by signs and symptoms, and may not interfere with daily activities. Therefore, it was also evident self-rated prevalence measurement is much more susceptible to underreport (56).

Many women are unaware of the unusual nature of their menstrual bleeding, especially in the developing world. They even think that there is no cure for it, so they do not take care to cope with it (57). In some cultural context and myths, excessive bleeding is one of the health signs (5). In some less developed countries in Southeast Asia, blood withdrawal is equal to purification (58). Previous studies have shown that 9% to 14% of women in reproductive age who experience menstrual bleeding of more than 80ml will ultimately undergo hysterectomy (59). The most important cause of hysterectomy in the United States and England has been abnormal uterine bleeding (4). The dysfunctions of menorrhagia and hypermenorrhea are associated with adverse effects on women such as anemia (60). 

If these menstrual disorders are prolonged and access to basic health care services is also limited, the severity of adverse effects on women will be more. In various research, menorrhagia is defined as bleeding of more than 80 ml per menstrual cycle. Previous studies revealed evidence that anemia is likely in bleeding less than 60 ml. in this case, taking iron supplements is not helpful if sever bleeding is not controlled, so Iron-level requirements cannot be met (61). In developing countries such as Iran, the priorities of the health system are the main causes of mortality and less attention is paid to morbidity (62). Recently, the global burden of disease, in addition to mortality, is also of particular interest to morbidity and quality of life (63). Although in developing countries, many studies have been done on menstrual disorders and their burden on the health system, these disorders continue to be neglected (64). Primary health services and sexual health programs in developing countries can increase their capacity to evaluate and provide services to women with menstrual disorders. Educational packages and easy therapies, like hormonal contraceptives, can be considered as commonly used facilities for detecting and preventing the disorders (65). 

The principal limitation of our study is disagreement between different studies on diagnostic criteria and existing definitions of menstrual disorders. In some studies, researchers have developed researcher tools and some others used standard and objective tools to determine the prevalence of the disorders. Another potential limitation was the high heterogeneity between studies. It can be due to the lack of uniformity of diagnostic criteria, populations, and sampling processes. In this review, we integrated the prevalence using a random effects model.

## Conclusion

Menstrual disorders are prevalent among Iranian women and associated with many adverse economic and social consequences. It has been neglected as a fundamental problem of women's reproductive health. Diagnosis and treatment of these disorders, especially in developing countries such as Iran, should be included in the primary health care system of reproductive health. Caregivers should be also trained to diagnose and treat disorders. 

## Conflict of interest

The authors declare that they have no competing interests.
